# Sex, offspring and carcass determine antimicrobial peptide expression in the burying beetle

**DOI:** 10.1038/srep25409

**Published:** 2016-05-03

**Authors:** Chris G. C. Jacobs, Sandra Steiger, David G. Heckel, Natalie Wielsch, Andreas Vilcinskas, Heiko Vogel

**Affiliations:** 1 Department of Entomology, Max Planck Institute for Chemical Ecology, Jena, Germany; 2 Institute of Evolutionary Ecology and Conservation Genomics, University of Ulm, Ulm, Germany; 3 Research Group Mass Spectrometry/Proteomics, Max Planck Institute for Chemical Ecology, Jena, Germany; 4 Institute for Insect Biotechnology, Justus Liebig University Giessen, Giessen, Germany

## Abstract

The burying beetle *Nicrophorus vespilloides* has emerged as a model system for the investigation of adaptations that allow the utilization of carrion as a diet and as a resource for reproduction. The survival of beetles and their offspring given their exposure to soil-dwelling and cadaver-borne microbes requires mechanisms that reduce bacterial contamination in the diet and that achieve sanitation of the microhabitat. To explore the role of antimicrobial peptides (AMPs) in this context, we analyzed burying beetle males and females at different stages of their breeding cycle using the RNA-Seq and proteomics approaches. To address variation in immune functions, we investigated the impact of adult sex, the presence or absence of offspring (social context), and the presence of carrion (environmental context) on the expression of the identified immune effector genes. We found that particular AMPs are sex-specific and tightly regulated by the presence of a carcass or offspring and identified the two most context-dependent antimicrobial proteins in anal secretions. The context-specific expression dynamics of particular AMPs and lysozymes reveals a complex regulatory system, reflecting adaptations to specific ecological niches. This study highlights how burying beetles cope with microorganisms found on carrion and identifies candidates for both internal and external immunity.

When an organism dies, an intense competition begins among vertebrate scavengers, invertebrate necrophores (including fly larvae and beetles), and bacterial and fungal decomposers, for the resources contained in its body. One of these invertebrate necrophores is the burying beetle (*Nicrophorus vespilloides*), which competes for access to small vertebrate carcasses to provide nutrition and resources for reproduction. Burying beetles reproduce by burying a small vertebrate carcass and molding it into a brood ball around which they lay their eggs^1^. Brood balls show little evidence of microbial decay and this has been attributed to antimicrobial compounds present in burying beetle oral and anal secretions^2^,^3^,^4^,^5^. Anal secretions of adult burying beetles contain more than 30 secondary metabolites and lysozyme-like factors with antimicrobial activity, which potentially contribute to the chemical preservation of the carcass^6^. Once the eggs have hatched, the larvae also contribute to the sanitation of the carcass^7^,^8^.

Burying beetles face strong competition from bacterial and fungal decomposers, which quickly rise in numbers on unattended carrion and reduce the quality of this breeding resource. To prevent the decrease in breeding resource quality they must have evolved counter strategies against carcass-derived microbes. Recently, we demonstrated that the exposure of experimentally isolated *N. vespilloides* eggs to soil-borne bacteria reduces the success of hatching because the eggs are not protected by endogenous antimicrobial compounds^9^. These and other observations inspired the hypothesis that the adult beetles invest in the protection of their offspring and that the context-specific expression of antimicrobial peptides (AMPs) may play a role in this process. Many insect species control the activities of microbes in their environment by producing AMPs, which can act synergistically at low concentrations^10^ and display a remarkable evolutionary plasticity reflecting adaptations to specific ecological niches^11^. AMPs play a key role in the innate immunity of insects exposed to pathogen-rich environments^12^,^13^,^14^, but their potential role in the internal (personal) immunity of the burying beetle and its external (secreted) immunity-related factors^15^ has not been explored in detail. However, recent studies provide evidence for the sex-specific expression of AMPs in beetles, providing the basis for context-dependent innate immunity^16^.

Here we combined transcriptomics (RNA-Seq) and proteomics (LC-MS^E^) to identify AMPs and lysozymes produced by the burying beetle. RNA-Seq analysis revealed the expression of genes encoding 27 putative AMPs and 13 lysozymes. To address context-dependent variation in immune functions, we investigated the impact of adult beetle sex, the presence or absence of offspring (social context), and the presence of carrion (environmental context) on the expression of these immune effectors (Fig. 1). Our experimental design allowed us to test the hypothesis that adult beetles specifically increase their investment in a parental form of social immunity^4^,^17^,^18^,^19^,^20^,^21^ if larvae are present, increasing larval survival^2^,^22^. The dynamic context-dependent expression of several immune effector genes supports their role in carcass preservation and provides new opportunities for the investigation of ecological immunology.

## Results

### Identification of AMPs and lysozymes

The screening of our *de novo* transcriptome assembly revealed 27 putative AMPs representing diverse families and functional classes. We identified one ABP, five members of the attacin family, one cecropin, five members of the defensin family, one KTX-like, three members of the PBSIP family, and six members of the thaumatin family (Supplementary Table S1). The search for lysozymes resulted in the identification of nine c-type lysozymes and four i-type lysozymes.

### Expression of AMPs and lysozymes in relation to carcass use

**Figure 1:**
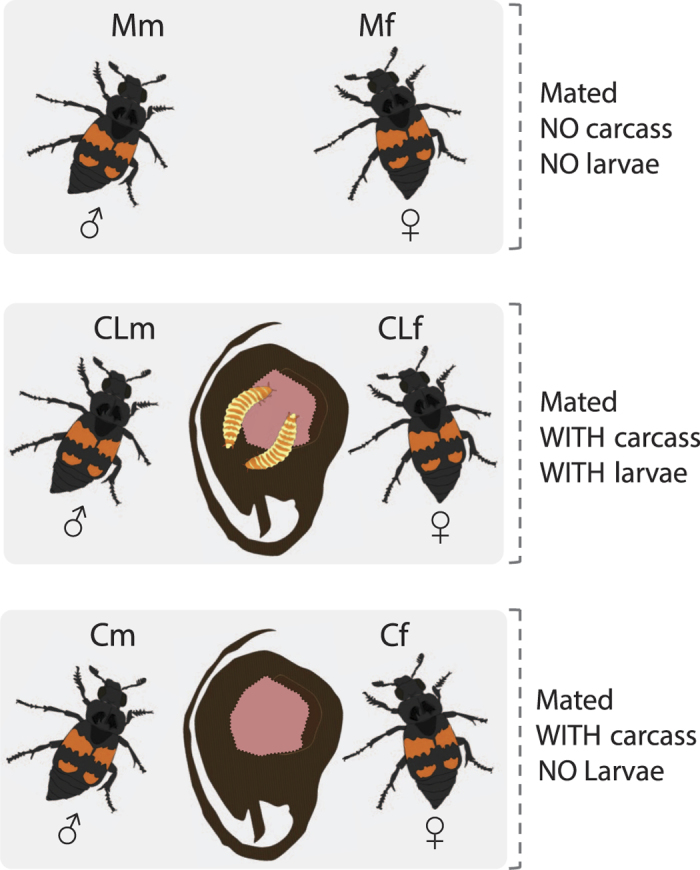
Overview of the experimental setup. Total RNA and anal secretions were collected from (1) males and females that were only allowed to mate (Mm, MF); (2) Mated males and females with carrion and larvae (CLm, CLf), and (3) mated males and females with carrion but without larvae (Cm, Cf).

**Figure 2:**
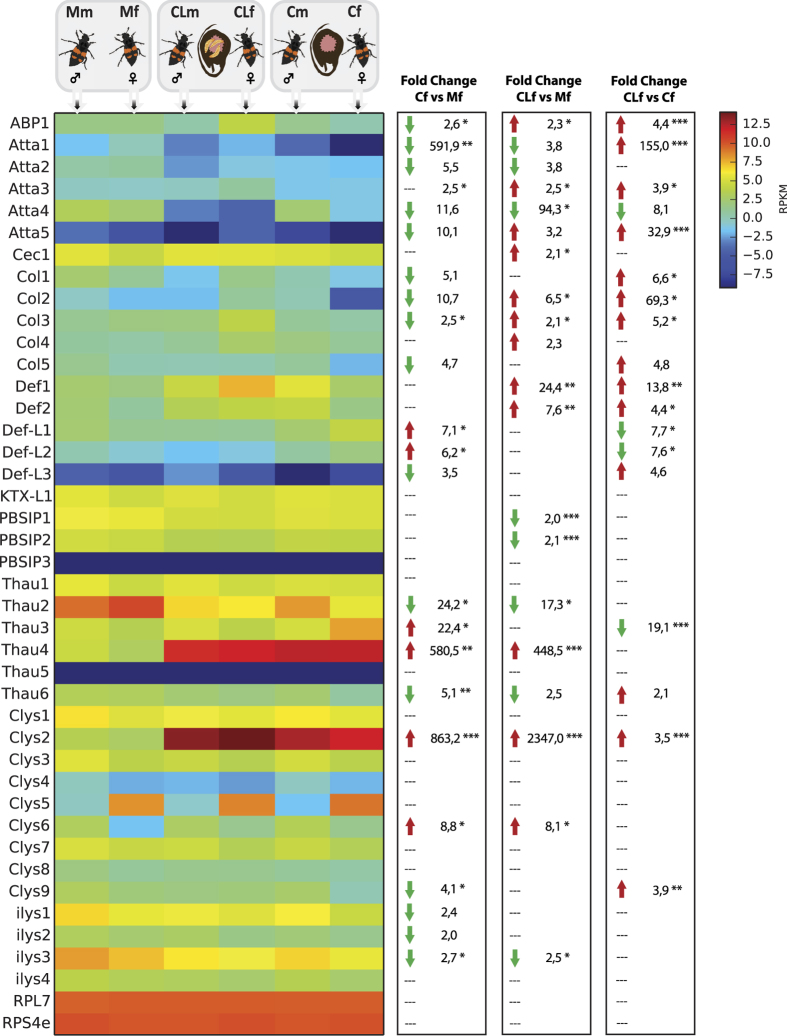
Heat map showing the relative expression levels of AMPs and lysozymes in males and females and in relation to social and environmental context. Significant differences for females are shown with an asterisk (* < 0.05, ** < 0.01, *** < 0.001). RPL7 and RPS4e are used as housekeeping genes and are shown to confirm the uniform expression of these control genes across tissues. The map is based on log2-transformed RPKM values (blue represents weakly-expressed genes, and red represents strongly-expressed genes).

We assigned the male (m) and female (f) beetles to three experimental groups (M, C, and CL), reflecting the presence or absence of carrion and differences in social context, i.e. the presence or absence of larvae (Fig. 1). Quantitative RNA-Seq analysis revealed the sex-dependent differential expression of AMPs as well as preferential expression in the presence or absence of larvae and of carrion (Fig. 2; Supplementary Table S2). Males and females presented with a carcass (Cm/Cf and CLm/CLf) expressed PBSIP1, thaumatin-2 and i-lysozyme-3 at lower levels than males and females with no carcass (Mm/Mf). In contrast, thaumatin-4 and c-lysozyme-2 were strongly induced in both sexes in the presence of a carcass (Fig. 2). Males also expressed lower levels of coleoptericin-1, PBSIP2 and thaumatin-6 but higher levels of defensin-1 and defensin-2 during carcass maintenance. The expression of c-lysozyme-6 increased in females maintaining a carcass. These data show that both AMPs and lysozymes are differentially expressed in relation to carcass use and could support the involvement of thaumatin-4 and c-lysozyme-2 in carcass preservation.

### Expression of AMPs and lysozymes in relation to parental care

Parental care provides a different social context to the presence of a carcass without larvae and such changes are also expected to influence the expression of AMPs and lysozymes. Accordingly, we observed extensive changes in gene expression, especially in females, when larvae were present. PBSIP2 was downregulated in both sexes. Attacin-4 was downregulated solely in males, whereas defensin-L1, defensin-L2, PBSIP1, thaumatin-2 and thaumatin-3 were downregulated solely in females. The expression of ABP1, cecropin-1, coleoptericin-1, coleoptericin-3, defensin-1, defensin-2, c-lysozyme-2, c-lysozyme-9 and i-lysozyme-1 was upregulated solely in females. Given that both sexes participate in parental care, it was surprising to find that only two AMPs and no lysozymes were modulated in males responsible for larval care, whereas 15 AMPs and lysozymes were modulated in females.

### Sex-dependent differences in AMP and lysozyme expression

Under all the conditions we tested, c-lysozyme-5 was expressed at much higher levels in females than in males, probably because it was expressed predominantly in the ovaries (unpublished data). In contrast, c-lysozyme-6 was expressed at consistently higher levels in males compared to females. In mated beetles without a carcass present, defensin-2, thaumatin-1, c-lysozyme-1, c-lysozyme-4 and c-lysozyme-6 were all expressed at higher levels in males. However, in the presence of a carcass without larvae, males expressed several c-type and i-type lysozymes, as well as attacin-4, thaumatin-2 and thaumatin-6, at higher levels than females, but females expressed higher levels of thaumatin-3. When actively feeding larvae were present on the carcass, several c-type and i-type lysozymes were expressed at higher levels in males whereas ABP1, coleoptericin-1 and defensin-1 were expressed at higher levels in females. Several lysozymes were generally expressed at higher levels in males compared to females, especially when maintaining a carcass and in the absence of larvae. The differences were small but statistically significant (Supplementary Table S2). In contrast, females expressed several AMPs at higher levels than males, and the differences were exacerbated by the presence of larvae.

### Identification of immune effector proteins in anal secretions

**Figure 3:**
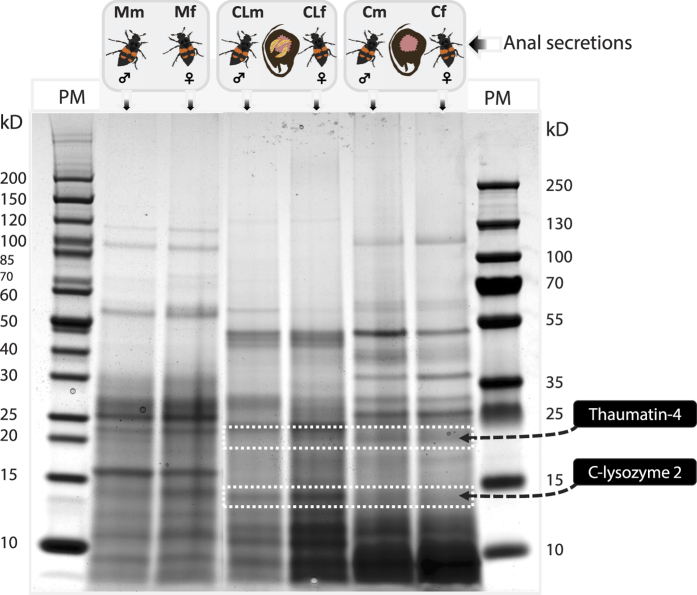
The protein composition of anal secretions from male (m) and female (f) beetles in the three experimental groups (M, C and CL) was investigated by SDS-PAGE. Lanes 1 and 8 show molecular size markers (PM) with molecular weights in kDa. Immunity-related protein bands identified by LC-MS^E^ are indicated by white boxes and arrows.

To confirm that the transcripts identified in the RNA-Seq dataset were also present as peptides or proteins in beetle anal secretions, we used a sensitive proteomics approach (LC-MS^E^) to detect c-lysozyme-2 and thaumatin-4, the most strongly expressed immune effectors. Anal secretions collected from male and female beetles in the three experimental groups (M, C, and CL) described above were resolved by SDS-PAGE. The proteins in the anal secretions displayed distinct banding patterns which differed between experimental groups, but were consistent between males and females of the same group (Fig. 3). The SDS-PAGE gel was cut into 20 bands representing different size fractions which were subsequently analyzed by LC-MS^E^. We identified peptides covering more than half of the predicted c-lysozyme-2 and thaumatin-4 protein sequences (Supplementary Figures S1 and S2, Table S3). We found that c-lysozyme-2 and thaumatin-4 were only detected in anal secretions of male and female C and CL beetles, but not in secretions of the M group without carrion and larvae (Fig. 3). Although for a number of AMPs moderate transcript levels in adults exposed to a carcass or attending larvae could be detected, only c-lysozyme-2 and thaumatin-4 proteins were identified in the anal secretions. There could be several reasons for this observation. The respective antimicrobial peptides might not be secreted into the gut lumen, or they may be secreted but subsequently degraded by proteases during passage though the gut. Alternatively, the AMPs may be present in anal secretions at levels below the detection threshold. Furthermore, proteolytic cleavage of AMPs can fail or be incomplete and it can be difficult to detect small peptides, especially those with amino acid repeats or disulfide bridges, resulting in the absence of specific peptide fragments for subsequent mass detection.

## Discussion

**Figure 4:**
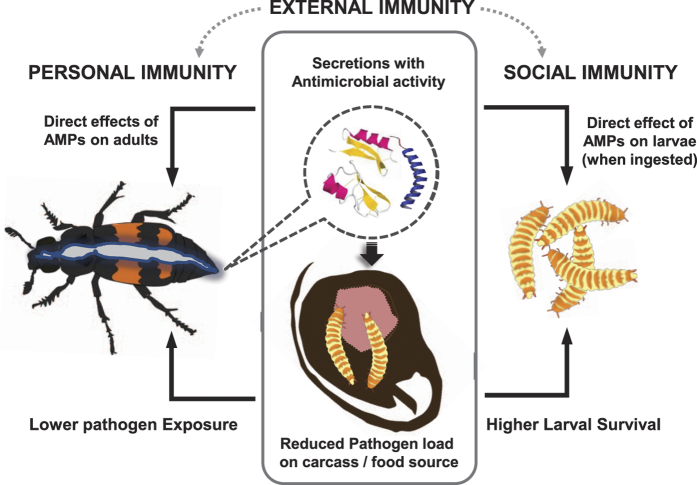
Proposed effects of anal secretions produced by burying beetles on social and personal (internal) immune function in adults and larvae. Shown are both potential direct and indirect effects of antimicrobials applied to carrion (external immunity).

When adult burying beetles prepare carrion, they both conserve and sanitize the animal carcass to suppress bacteria-mediated decay and fungal growth^2^,^3^,^4^,^7^,^8^. Because contact with carrion exposes the beetles to microbes, this environmental sanitation not only benefits the larvae but also adults, directly by minimizing the microbial load present in their own food source and indirectly by increasing the survival of their offspring. This dual role of environmental sanitation, mediated by burying beetle secretions, blurs the distinction between personal and social immunity (Fig. 4). Although the carrion is sanitized, carrion flesh consumption nevertheless presents an ongoing risk to increase the ingestion of potential pathogenic microbes by the adults, and therefore the expression of AMPs and lysozymes is anticipated. AMPs and lysozymes could contribute to both the internal and external immunity of the beetles, i.e. by controlling the growth of ingested microbes as well as preserving the carcass (Fig. 4)^15^.

Accordingly, our RNA-Seq approach extended the panel of known burying beetle AMPs from the four originally reported^23^ to 27 putative AMPs and 13 lysozymes, suggesting that immunity-related effector genes have expanded in this species more than in the model beetle *Tribolium castaneum* but less than in the harlequin ladybird *Harmonia axyridis*^24^,^25^. Several lysozymes displayed both sex-dependent and social context-dependent expression profiles. The strongest effect was observed for c-lysozyme-2, which was expressed at higher levels than the reference genes when carrion was present (Fig. 2). These findings make c-lysozyme-2 a primary candidate for at least part of the lytic activity observed in burying beetle anal exudates^2^ and are in line with recent work describing the high expression of a single lysozyme in female guts when breeding^26^. Contrary to the 6 C-lysozyme sequences that have been reported by Palmer *et al*.^26^, we have identified 9 C-type lysozyme sequences. This difference is likely due to the fact that we have analyzed whole bodies of males and females, not limiting AMP and lysozyme discovery to only those genes expressed in the female gut.

We furthermore observed a strong female-specific bias for the expression of c-lysozyme-5, which is expressed in the ovaries (unpublished data). Given that burying beetle eggs are susceptible to soil-borne bacteria, but show no endogenous immune responses^9^, this lysozyme might be essential for egg survival. Interestingly, it has been shown in another beetle that c-type lysozymes synergize with other AMPs such as coleoptericins^27^.

Many of the differentially expressed AMPs we identified correlated with the presence or absence of carrion and/or larvae (Fig. 2 and Supplementary Table S1). However, some members of each AMP family were upregulated in the presence of carrion whereas others were downregulated under these conditions, indicating that there is no strict assignment of particular roles to individual families. The downregulation of several AMPs in relation to carrion use indicates a functional divergence between (at least part of) the AMPs involved in internal and external immunity. Immune responses are costly in terms of energetic resources or nutrient availability^28^,^29^,^30^,^31^,^32^, so trade-offs between different immune strategies are anticipated^33^. However, although such trade-offs and differential investments of male and female *N. vespilloides* have been proposed^5^,^20^,^21^, we could not identify a clear pattern suggesting overall higher investment in AMP production in female burying beetles. Similarly, in *N. orbicollis* both males and females showed increased encapsulation rates and higher lytic activity in their anal exudates when presented with a carcass^18^. Therefore, the simultaneous investment in personal and social immunity that normally involves a competition for resources may be an adaptive response in species such as the burying beetle that breed in microbe-rich environments^18^ (Fig. 4). Although little is known about the personal immune responses of burying beetles, our data provides the basis for dissecting the effectors of both immune strategies in this species.

The strong induction of thaumatin-4 in adult beetles exposed to carrion suggested that lysozyme is not the only protein important for the antimicrobial properties of the secretions. This hypothesis was supported by the identification of c-lysozyme-2 and thaumatin-4 proteins in the anal secretions of adult *N. vespilloides*, representing immune effectors that are likely to be active against bacteria^34^ and fungi^35^, respectively. These immune effectors could be detected in both males and females only when carrion was present, irrespective of the presence or absence of larvae, indicating that the presence of carrion and not of larvae is a major determinant of expression of those AMPs found in anal secretions. Although exposure to a different food source during carrion utilization could explain some of the gene expression changes we observed, the identification of these immune effector proteins directly in the anal secretions of adults is strong evidence for their key role in carrion sanitation.

The diversity of bacteria and fungi encountered on carrion probably requires a range of different antimicrobial compounds with overlapping activities in burying beetle secretions, and this challenge could be met not only by increasing the spectrum of antimicrobial products but also by exploiting the synergies that occur among coexpressed insect AMPs and lysozymes^27^,^36^. It has previously been suggested that a single lysozyme is the key effector molecule for social immunity in *Nicrophorus*^26^, even though the respective protein was not identified in the anal exudates of the beetles. Although our data indicate that the antimicrobial effects of anal secretions are mediated primarily by two major immune effector proteins, functional interactions among AMPs expressed at much lower levels^10^,^36^ could increase the number of candidates for external immune defense in the burying beetle. Future studies detailing the contributions of each single component of the secretions and their antimicrobial properties will shed light on the relative importance of each component for the overall activity of the secretions.

Our study of the immune repertoire of the burying beetle clearly identifies prime candidates for carrion sanitation and provides a basis for more detailed studies exploring the interactions between insect and microbes, both in the context of social and personal immunity. We have also provided evidence for the context-specific expression of particular AMPs and lysozymes according to sex, the presence of offspring and the presence of a carcass, revealing a complex system of transcriptional reprogramming reflecting adaptations to specific ecological niches. Because immune responses are costly, the strictly context-dependent regulation of individual AMPs may reduce the penalty associated with the reallocation of resources from other fitness-related traits such as fecundity.

## Methods

### Experimental procedure

The third-generation male (m) and female (f) offspring of burying beetles captured in a deciduous forest in Ulm, Germany, were assigned to three experimental groups (M, C, and CL) reflecting the presence or absence of carrion and differences in social context, i.e. the presence or absence of larvae (Fig. 1). Mm and Mf represent mated males and females without carrion or larvae. A single male and a single female were kept together in a plastic container (10 × 10 × 6 cm) filled with moist peat for 24 hours. Cm and Cf represent unrelated males and females with carrion but without larvae, whereas CLm and CLf represent unrelated males and females with carrion and larvae. A single male and a single female were kept together in a plastic container as above, filled to half-depth with moist peat and also provided with a 10 g mouse carcass. When the carcass was buried, the containers were kept in darkness and subsequent steps were carried out under red light. After 48 h, the adult beetles and their carcass were transferred to a new container. A small piece of mouse carcass was added to the original container because the larvae crawl towards the carrion immediately after hatching, allowing us to determine the time when a pair’s larvae began to hatch. Embryonic development takes ~56 h at 20 °C^37^. We first checked for the presence of larvae 64 h after the adults had been provided with a carcass and every 8 h thereafter. As soon as the first larva was observed, their respective parents were transferred to a new box. In the CL treatment group, a standardized number of 10 first-instar larvae were placed on the carcass to be fed by the adults. In the C treatment group, no larvae were provided. After a further 24 h (day 23 of the adult phase) all the parent beetles were freeze-killed. Therefore, all the C and CL beetles had been exposed to a carcass for the same amount of time, but only the beetles representing the CL group had cared for larvae for 24 h.

### RNA-Seq, *de novo* assembly and candidate gene identification

Total RNA was isolated from individual male and female beetles in each of the three treatment groups. We collected three biological replicates per sex and per treatment. RNA was isolated according to the manufacturer’s instructions (innuPREP RNA Mini Kit, Analytik Jena, Jena, Germany). Transcriptome sequencing was carried out for 18 different whole-adult RNA samples using poly(A)^+^ enriched RNA fragmented to an average of 150 nucleotides. Sequencing was carried out by the Max Planck Genome Center Cologne (MPGCC) on an Illumina HiSeq2500 Genome Analyzer platform using paired-end (2 × 100 bp) reads. This yielded approximately 30 million reads for each of the 18 samples. Quality control measures, including the filtering of high-quality reads based on fastq file scores, the removal of reads containing primer/adapter sequences, and trimming of the read length, were carried out using CLC Genomics Workbench v7.1 (http://www.clcbio.com). The same software was used for *de novo* transcriptome assembly, combining all 18 RNA-Seq samples, and selecting the presumed optimal consensus transcriptome as previously described^37^. The final *de novo* reference transcriptome assembly (backbone) of *N. vespilloides* contained 55,918 contigs (minimum contig size = 200 bp) with an N50 contig size of 1271 bp and a maximum contig length of 24,850 bp. The transcriptome was annotated using BLAST, Gene Ontology and InterProScan searches implemented in BLAST2GO PRO v2.6.1 (www.blast2go.de) (Götz *et al*. 2008) as previously described^38^. To identify candidate AMP and lysozyme genes expressed in *N. vespilloides*, we established a reference set of known or predicted insect-derived AMPs and lysozymes using published sequences and by searching our in-house database as well as public databases (NCBI). In order to avoid interpreting incomplete genes or allelic variants as different AMP genes, we have implemented a number of additional filters to obtain a more conservative number of candidate AMP genes. We manually curated all of the candidate AMP and lysozyme sequences using the following criteria: i) all AMP candidate sequences were translated into the respective amino acid sequence and multiple alignments were performed with all members of a specific AMP family. Based on the alignments we removed sequences which covered less than 50% of the coding region as well as redundant sequences, defined as those sequences displaying zero or only a single amino acid difference. We then inspected all sequences which displayed only few amino acid differences (e.g. two amino acid differences observed between coleoptericins 2 and 5) and assessed contig sequence differences at the nucleotide level. AMPs were assigned to different genes only where the total number of nucleotide differences exceeded the number of nucleotide changes resulting in differences at the amino acid level. Although we think that we have been conservative in our definition of different AMP genes, we cannot completely rule out the possibility that some allelic variants could also display greater differences at the nucleotide level.

### Mapping and detection of differential expression

Digital gene expression analysis was carried out using CLC Genomics workbench to generate BAM mapping files, QSeq (DNAStar Inc.) to remap the Illumina reads from all 18 samples onto the reference transcriptome, and finally by counting the sequences to estimate the expression levels, using previously described parameters for read mapping and normalization^38^. Briefly, for the read mapping we have used the following parameters: read assignment quality options required at least 50% of the total read bases (how much of the sequence should be able to map in order to include it) and at least 90% of bases matching (minimum similarity fraction; defines how exact the matching part of the read should be) within each read to be assigned to a specific contig; maximum number of hits for a read (reads that match to more distinct places than this number will not be mapped) =10; mer repeat settings were automatically determined while other settings were not changed. Biases in the sequence datasets and different transcript sizes were corrected using the RPKM algorithm (reads per kilobase of transcript per million mapped reads) to obtain correct estimates for relative expression levels. To control for the effect of global normalization using the RPKM method, we also analyzed a number of highly-conserved housekeeping genes, including several genes encoding ribosomal proteins (Rpl3, Rpl5, Rpl7, Rps4e, Rps5, Rps8, Rps18, and Rps24), NADH-dh, elongation factor 1α and eukaryotic translation initiation factors 4 and 5. The overall variation of expression levels for these housekeeping genes across samples and treatments was lower than 1.3-fold (based on log2 transformed RPKM values), indicating they were not differentially expressed. Because RNA-Seq data are noisier at lower expression levels, we excluded genes for which log2 (RPKM) was less than 1 under any of our experimental conditions. RPL7 and RPS4e were used as reference genes and are shown in the heat map (Fig. 2) to confirm the similar expression of control genes across tissues. The log2 (RPKM) values (normalized mapped read values; geometric means of the biological replicate samples) were subsequently used to calculate fold-change values. To identify differentially expressed genes, we have used Student´s t-test (as implemented in Qseq) corrected for multiple testing using the Benjamini–Hochberg procedure to control the false discovery rate (FDR). The differential expression (fold-change values) of the AMP and lysozyme genes, and the statistical significance thereof (Student’s t-test; FDR-corrected p-values), are summarized in Supplementary Table S1. Predicted AMP and lysozyme sequences are listed in Supplementary Table S2.

### Identification of proteins in anal secretions

The precise identification of AMP and lysozyme candidate proteins based on our transcriptome dataset was achieved using the MS-E approach. Using a pipet tip, anal secretions were collected from *N. vespilloides* males and females from different treatment groups as described above. Anal exudates can easily be obtained by handling the beetles, which usually provokes exudation of the brown liquid from the anus. The exudates were mixed with three volumes of phosphate-buffered saline (PBS) containing 1× Pierce protease inhibitor cocktail and stored at −80 °C. The samples were thawed on ice and centrifuged at 10,000 *g* for 5 min to pellet any particulate matter, then boiled to denature the proteins and loaded onto a Criterion 4–12% polyacrylamide gradient SDS-PAGE gel (BioRad, Hercules, California). After separation, the gel was stained with Coomassie Billiant Blue. 20 segments representing different protein size ranges were excised and digested with trypsin. The tryptic peptides were extracted as previously described^39^ and reconstructed in 10 μL aqueous 0.1% formic acid for LC-MS^E^ analysis using an Acquity nano-UPLC system connected on-line to a Q-ToF Synapt HDMS mass spectrometer (Waters, Milford, USA). Predicted peptide masses were used to search the *Nicrophorus* AMP and lysozyme protein sub-database (see Supplementary Materials for details).

## Additional Information

**How to cite this article**: Jacobs, C. G. C. *et al*. Sex, offspring and carcass determine antimicrobial peptide expression in the burying beetle. *Sci. Rep*. **6**, 25409; doi: 10.1038/srep25409 (2016).

## Supplementary Material

Supplementary Information

Supplementary Table S1

Supplementary Table S2

Supplementary Table S3
