# Supplementary material for: Sex, offspring and carcass determine antimicrobial peptide expression in the burying beetle
Source: Sci Rep. 2016 May 3;6:25409. doi: 10.1038/srep25409 (PMC4853764; doi:10.1038/srep25409)
Supplement: Supplementary Information [file srep25409-s1.pdf]

Supplementary Material

Sex, offspring and carcass determine antimicrobial peptide expression in the burying beetle

Chris G.C. Jacobs, Sandra Steiger, David G. Heckel, Andreas Vilcinskas and Heiko Vogel

Supplementary Methods

Identification of proteins in anal secretions

To enable a precise identification of AMP and lysozyme candidate proteins utilizing our transcriptome database, we used an MS-E approach. Anal secretions from *N. vespilloides* males and females from different treatment groups as described in the Main text were collected using a pipet tip. Anal exudates can easily be obtained by handling the beetles, which usually causes them to release the brown liquid from the anus. We mixed anal exudates collected and pooled from four individuals each with phosphate-buffered saline (PBS) containing protease inhibitor cocktail (1x, Pierce) in a 1:3 ratio (V/V) and stored the mixtures in a -80 °C freezer until further processing. Before resolving the proteins on SDS-PAGE gels, samples thawed on ice were centrifuged at 10,000 g for 5 minutes to pellet any particulate matter. Heat denatured protein samples were separated on a Criterion 4-12 % gradient polyacrylamide SDS-PAGE gel (BioRad) and stained using Coomassie blue. Twenty different size sections were excised from the Coomassie-stained gel and tryptic digestion and extraction of tryptic peptides from gel pieces was carried out as described before[1]. For LC-MS, analysis samples were reconstructed in 10 µL aqueous 0.1 % formic acid. The samples were analyzed using a nano Acquity nano-UPLC system on-line connected to a Q-ToF Synapt HDMS mass spectrometer (Waters, Milford, USA). Desalting of samples was performed using a Symmetry C18 trap-column (20 x 0.18 mm, 5 µm particle size, Waters, Milford, USA) at a flow rate of 15 µl/min followed by peptide separation on a nano Acquity C18 analytical column (200 mm ×75 µm ID, C18 BEH 15 130 material, 1.7 µm particle size, (Waters, Milford, USA)). Mass spectrometer settings were as described previously[2]. LC-MS data were acquired in positive ESI mode under data-independent acquisition (MSE) controlled by MassLynx v4.1 software. The collision energy was set at 4 eV in low energy (MS) scans, and ramped from 15 to 40 eV in elevated energy (MSE) scans. The mass range (m/z) for both scans was 300–1900 and 50–1700 Da, respectively. The scan time was set at 1.5 sec for both modes of acquisition with an inter-scan delay of 0.2 sec. A reference compound, human Glu-Fibrinopeptide B (650 fmol/ml in 0.1% formic acid/acetonitrile (v/v, 1:1)), was infused continuously through a reference sprayer for external calibration. ProteinLynx Global Server (PLGS) version 2.5.2 (Waters, Milford, USA) was used for processing of raw files and for database searching. The continuum

LC-MSE data were lock-mass-corrected, smoothed, background-subtracted, centered, deisotoped, and charge-state-reduced. Thresholds for low/high energy scan 5 ions and peptide intensity were set at 150, 30, and 750 counts, respectively. Processed data were searched against the Swissprot database (downloaded on December 14, 2014, from http://www.uniprot.org/) combined with *N. vespilloides* AMP and lysozyme protein sub-database constructed from *N. vespilloides* transcriptome database by their translation from all six reading frames. Database searches were performed at 2% false discovery rate (FDR), using the following parameters: minimum number of product ion matches per peptide (5), minimum number of product ion matches per protein (7), minimum number of peptide matches (2), and maximum number of missed tryptic cleavage sites (1). Details of the identified peptides can be found in Supplementary Table S3.

References

1. Shevchenko, A., Tomas, H., Havlis, J., Olsen, J.V. & Mann, M. In-gel digestion for mass spectrometric characterization of proteins and proteomes. *Nat. Protoc.* **1**, 2856-2860 (2006).

2. Kirsch, R., Wielsch, N., Vogel, H., Svatos, A., Heckel, D. G. & Pauchet, Y. Combining proteomics and transcriptome sequencing to identify active plant-cell-wall-degrading enzymes in a leaf beetle. *BMC Genom* **13**, 587 (2012).

Supplementary Figures

MKSALVFGLLFLGAAHCAQFRVKNVMGGDIWIGILTSNNLPSLEGGGFVLGPQQER**AINAPDDWEGR**FW
AR**TWCNSGSQHCETGDCGNK**VQCNGAGGVPPASLAEFNLK**AWNDGKDYYDISLVDGYNVGVKIEPLGG**
SGDCNVLHCSNNLNDNCPNELRK**YGSGGTIACESSCNKFDK**DEYCCR**NEFNDPKICNPNTWAVNSAKYFK**
DNCPDAYSYAYDDHSSLKTCMAGTYRITFG

Fig. S1. The peptide sequence coverage map for *N. vespilloides* Thaumatin-4 protein. The peptides recovered from the MS analysis are listed and mapped onto the protein sequence. Regions of the protein sequence that match MS-E identified peptides are highlighted in blue. The predicted signal peptide is depicted in red.

MKFAICAIVLCGLVAPNFAKVFSPTEFGNVMLANGIPK**GQIETWKCIAKWESSYNTAAHNTASGDHGIFQI**
NERWWCSPPGKGCGMTCNSLRDADITNDIKCAK**IIYEEHQRLTGNGFNAWVAYK**NHCR

Fig. S2. The peptide sequence coverage map for *N. vespilloides* C-Lysozyme-2 protein. The peptides recovered from the MS analysis are listed and mapped onto the protein sequence. Regions of the protein sequence that match MS-E identified peptides are highlighted in blue. The predicted signal peptide is depicted in red.
